# Supplementary material for: Evaluation of SSTR2 Expression in SI-NETs and Relation to Overall Survival after PRRT
Source: Cancers (Basel). 2021 Apr 23;13(9):2035. doi: 10.3390/cancers13092035 (PMC8122794; doi:10.3390/cancers13092035)
Supplement: Supplementary file 1 [file cancers-13-02035-s001.zip › cancers-1158118_Supplementary_Final.pdf]

Supplementary Materials

Evaluation of SSTR2 Expression in SI-NETs and Relation to Overall Survival after PRRT

Anna-Karin Elf, Viktor Johanson, Ida Marin, Anders Bergström, Ola Nilsson, Johanna Svensson, Bo Wängberg, Peter Bernhardt and Erik Elias

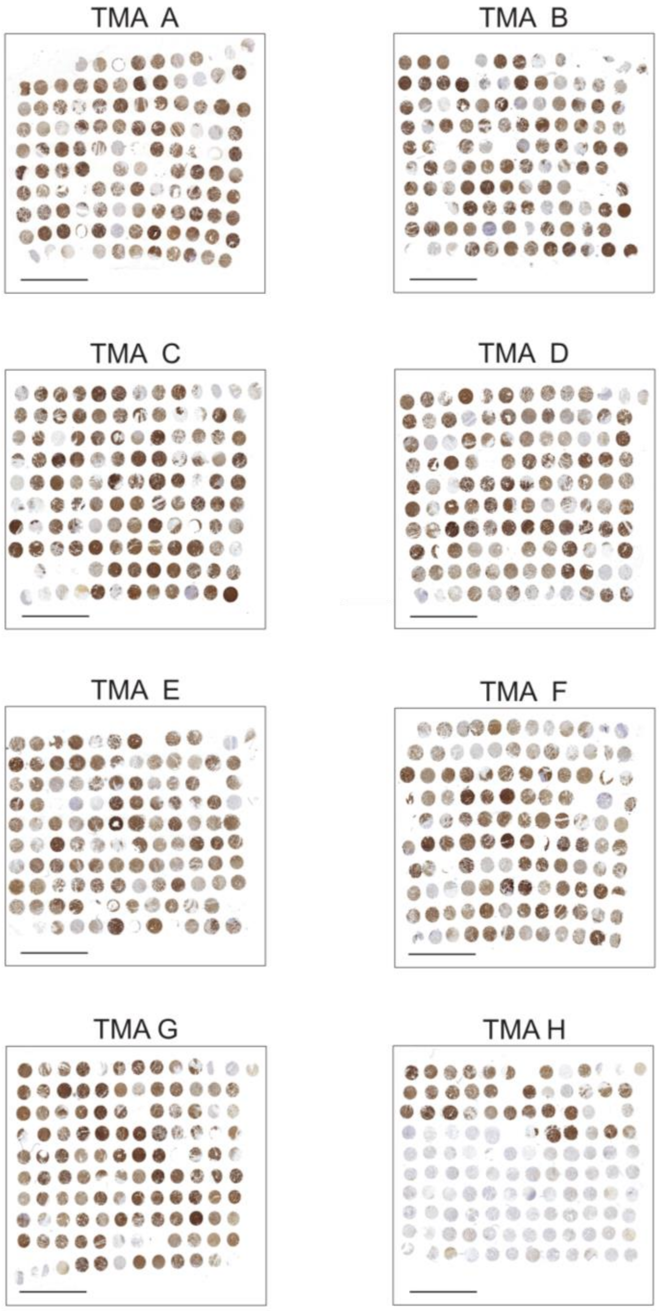

Figure S1. SSTR2 stained TMA blocks. Size bar = 5mm.

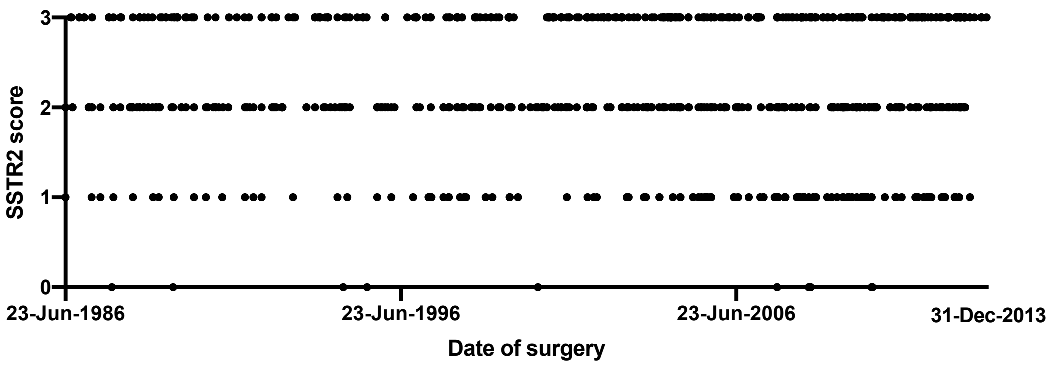

Figure S2. Distribution of SSTR2 score according to date of surgery. Each point represents one sample on the TMA (see Suppl fig 1). Correlation analysis was performed for all samples on the TMA. There was no significant correlation, which argued against age of samples as factor determining SSTR2 score. r = -0,01026, p = 0,7736. Spearman rank correlation.

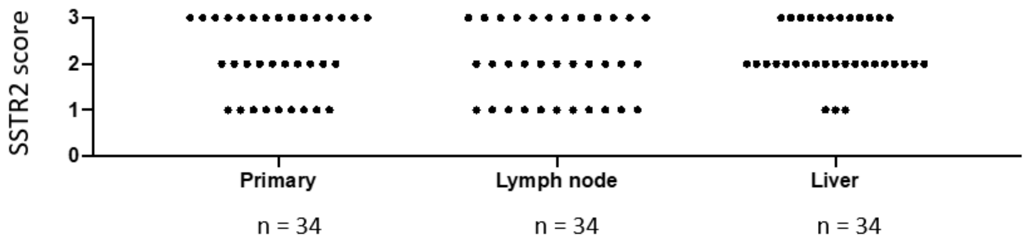

Figure S3. Distribution of SSTR2 score according to tumor site. SSTR2 score did not vary significantly between tumor sites (p = 0.52; Kruskal-Wallis test). Each point represents one sample.

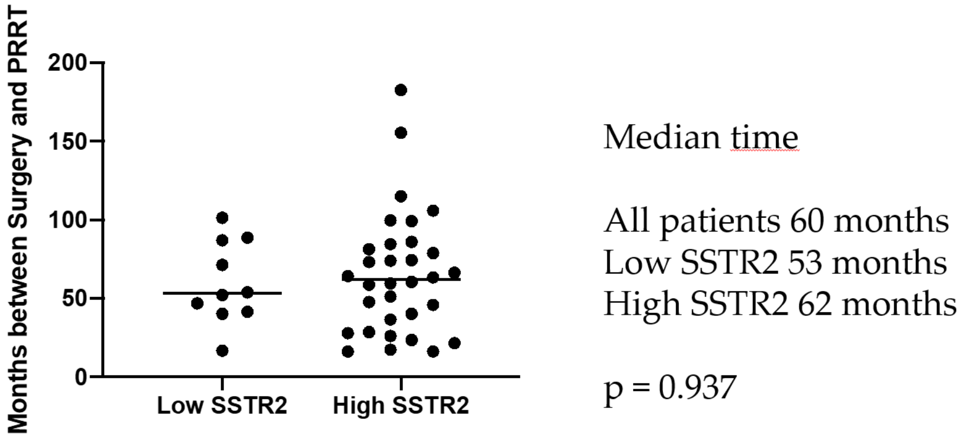

Figure S4. Time between surgery and PRRT. Each point represents one patient. There was no significant time difference between the patient groups "Low SSTR2" (n=10) and "High SSTR2" (n=32). Lines show median. Mann-Whitney U test.
